# Real-time detection of extravasation events of diagnostic and therapeutic radiopharmaceuticals in oncology

**DOI:** 10.1371/journal.pone.0350116

**Published:** 2026-06-01

**Authors:** Noemi Cucurachi, Federica Fioroni, Elisa Grassi, Nicola Panico, Laura Verzellesi, Andrea Botti, Alessandro Fraternali, Massimo Roncali, Rexhep Durmo, Angelina Filice, Mauro Iori

**Affiliations:** 1 Medical Physics Unit, Azienda USL-IRCCS di Reggio Emilia, Reggio Emilia, Italy; 2 Medical Physics Unit, Azienda Socio Sanitaria Territoriale di Mantova, Mantova, Italy; 3 Department of Physics, University of Bologna, Bologna, Italy; 4 Nuclear Medicine Unit, Azienda USL-IRCCS di Reggio Emilia, Reggio Emilia, Italy; Ural Federal University named after the first President of Russia B N Yeltsin Institute of Physics and Technology: Ural’skij federal’nyj universitet imeni pervogo Prezidenta Rossii B N El’cina Fiziko-tehnologiceskij institut, RUSSIAN FEDERATION

## Abstract

**Background:**

Radiopharmaceutical extravasation—when injected activity remains in soft tissue rather than entering the bloodstream—can compromise quantification in positron emission tomography/computed tomography (PET/CT) and, in therapeutic settings, deliver high local radiation doses. These effects may lead to inaccurate Standardized Uptake Value (SUV) measurements, potential misdiagnosis, repeat examinations, and to avoid patient risk. We assessed a simple, real-time approach for detecting extravasation during administration to enable immediate management and quantitative correction.

**Results:**

Real-time monitoring with portable gamma detectors was applied to 885 diagnostic PET examinations (^18^F-FDG, ^68^Ga-DOTATOC/TATE, ^68^Ga-PSMA-11) and 15 administrations of ^177^Lu-DOTATATE therapy. In the diagnostic cohort, 53 extravasation events were identified and confirmed on PET images. Analysis of dose-rate–time curves yielded practical thresholds for extravasation detection: Δp^in^_nor_ = 0.30 and ΔR_t_ = 388 µSv/h. A statistically significant association was observed between SUV correction factors and specific curve parameters, enabling prospective adjustment of SUV before image interpretation. In the therapeutic cohort, one extravasation was observed, with an estimated absorbed self-dose of 11.6 Gy to the affected region. The monitoring enabled timely intervention that limited unnecessary exposure and workflow disruption. Feasibility analyses indicated that, for diagnostic procedures, a single detector can be sufficient for reliable extravasation identification and for deriving SUV correction factors.

**Conclusions:**

A real-time, detector-based strategy can identify radiopharmaceutical extravasation during injection, support immediate mitigation, and preserve diagnostic accuracy by allowing early SUV correction. In therapeutic applications, it can reduce risk by prompting management of rare but consequential events. The operational simplicity, low cost, and effectiveness demonstrated across both diagnostic and therapeutic scenarios support integration of this approach into routine clinical practice, with the added practicality that a single detector appears adequate for diagnostic workflows.

## Introduction

Nuclear medicine therapy and diagnostic procedures use radiopharmaceuticals, administered through intravenous injection (IV) or capsules. The administration procedures require the establishment of robust clinical services to ensure both efficacy and patient safety [1–4].

Radiopharmaceutical extravasation events can occur during IV administration, both in therapy and diagnostic procedures. An extravasation occurs when some fraction of radiopharmaceutical remains in the soft tissue area close to the injection site, rather than the bloodstream, due to improper placement of the IV access device or vessel wall failure [5,6]. Because extravasation may result in a large local radiation dose to the tissue [18], avoiding these events is crucial, as undue absorbed doses at the injection site can adversely impact study results and diagnostic procedures [7]. Indeed, it may be necessary to repeat a diagnostic examination or administer an additional therapeutic dose. Extravasation can also lead to local damage, such as rash or epithelial necrosis, especially in treatments. Although the consequences of extravasation events are considered more severe with therapeutic radiopharmaceuticals (beta or alpha emitters) 7], even in diagnostic activities extravasation can compromise the accuracy of quantitative parameters essential for clinical diagnosis [8–13].

Identifying an extravasation event during diagnostic procedures, such as positron emission tomography/computed tomography (PET/CT) examinations, can prevent department organisation problems related to the necessity of repeating the diagnostic procedure leading to radioprotection issues due to re-injection of the radiopharmaceuticals. Also, the presence of an extravasation region introduces various confounding factors during diagnostic procedures including misidentification of lesions, classification of scans as non-diagnostic, and underestimation of Standardised Uptake Values (SUV) by 19–73% [19]. In particular, PET/CT images are usually obtained in the arms-up position, if an extravasation event occurs, the arms can cause artifacts regardless of up or down positioning [24]. In consideration of SUV underestimation, it is a critical issue in interpreting Positron Emission Tomography (PET) exams. This parameter is widely used for diagnosis, staging, and therapy response evaluation in nuclear medicine, but it is susceptible to variability due to physical and biological factors, suboptimal image acquisition, and processing [8].

The growing awareness of avoiding tissue doses in case of therapeutic or diagnostic extravasations and accurately quantifying SUV has prompted increased attention to radiopharmaceutical extravasation events. The commercialization of the LARA device, equipped with topically applied scintillation sensors generally placed on the two arms, has facilitated the monitoring of the injection process and identification of extravasation occurrences [14–18]. This method inspired the group of Perrin [25] that proposed a method to classify extravasation events using the curves acquired on injection and contralateral arms using LARA topical detectors. The group of Mazzara C. et al [26] proposed a mathematical model for real-time detection and characterization of ^177^Lu-DOTATATE extravasation events using a survey meter. They acquired equivalent dose rate data, monitoring the signal 20 cm from the patient’s arm and abdomen, and modeling the infusion process.

Our work introduces a novel method for monitoring diagnostic radiopharmaceutical administration, accompanied by the metrics presented in our previous paper [23] to characterize and classify the administration process. We have expanded our work by carrying out monitoring during radioligand therapy with ^177^Lu-DOTATATE.

We have therefore acquired ^177^Lu-DOTATATE curves to characterise therapeutic injections trying to find a metric that fits this type of curve’s trend. Our goal is to propose an operationally simple and efficient method for detecting the presence of extravasation, characterizing it, and, in the case of therapeutic injections, halting the injection process. In diagnostic cases, we aim to estimate the SUV correction coefficient (SUV_corr,coeff_) to apply to SUV calculated on diagnostic images by nuclear medicine physicians and decide if a re-injection is necessary before the images are acquired.

In comparison to the previous publication [23], we continued our study by collecting more data from patients administered diagnostic radiopharmaceuticals (^18^F-FDG, ^68^Ga-DOTATOC/TATE, and ^68^Ga-PSMA-11) and patients treated with a therapeutic radiopharmaceutical (^177^Lu DOTATATE), aiming to achieve greater statistical significance and to verify the applicability of the method to therapeutic cases as well. In contrast to existing procedures, our system enables the rapid identification of abnormal/extravasation events, so it can be clinically applied by healthcare personnel, allowing for the timely identification of extravasation, and providing an estimate of the correction factor necessary for accurate SUV evaluation. Also, our approach allows remote and distance monitoring, minimizing exposure for the involved staff.

## Materials and methods

### Study cohort

The study was conducted in accordance with the Declaration of Helsinki and approved by the Ethics Committee of the Area Vasta Emilia Nord (approved on 22/06/2021 with registration number 448/2021/SPER/IRCCSE). The study enrolled 885 patients undergoing ^18^F-FDG, ^68^Ga-DOTATOC/TATE/PSMA-11 PET examinations, and 15 patients undergoing ^177^Lu-DOTATATE therapies. In accordance with the procedure approved by the ethics committee, all patients provided informed written consent for the conduction of the study and the publication of scientific data before the radiopharmaceutical injection for PET studies and for ^177^Lu-DOTATATE therapies. The cohort of patients was enrolled between 03/08/2021 and 15/07/2025.

The patients’ ages involved in this study were in the range of 17–90 years, with a median of 63 years. The injected activities have a median value with an interquartile range equal to 187 [161–215] MBq for diagnostic radiopharmaceuticals, injection activities of ^177^Lu-DOTATATE are in a range of 7100–7400 MBq.

Patients who weren’t in good health, had limited mobility, or presented clinical conditions unsuitable for proper monitoring during radiopharmaceutical administration were excluded from the study. These exclusions were necessary to ensure the accuracy and reliability of the monitoring process. Additionally, we systematically recorded a range of clinical parameters that could potentially influence the intravenous (IV) administration procedure. These included demographic factors such as age, weight, and height, as well as physiological and medical factors like glucose levels and ongoing treatments with corticosteroids or chemotherapy. The inclusion of these variables allowed us to investigate their potential impact on radiopharmaceutical administration outcomes.

To explore possible correlations between these clinical factors and the acquisition parameters of dose rate (DR)-time curves, we conducted a statistical analysis using the Pearson correlation test. A p-value threshold of < 0.05 was considered statistically significant, indicating meaningful associations between specific parameters and deviations in radiopharmaceutical administration. This analysis aimed to identify any underlying patterns that could influence the effectiveness or accuracy of the procedure.

### Instruments and equipment

Patient monitoring was performed using a personal gamma spectrometric radiation detector RadEye SPRD-ER and two personal gamma dosimetric radiation detectors RadEye PRD-ER of Thermo Fisher Scientific (Waltham, Massachusetts, USA).

The SPRD-ER detector incorporates two highly sensitive scintillation detectors, a CsI(TI) for low dose rates and PVT for high dose rates, instead, PRD-ER incorporates NaI(TI) with a miniature photomultiplier for the detection of very low radiation levels. They are calibrated in ambient dose equivalent H*(10) [µSv/h] and their sensitivity energy range (40KeV - 3MeV) is suitable for monitoring diagnostic and therapeutic radiopharmaceuticals. They are portable devices and can be wearable thanks to their low weight (0.16 kg). The devices are equipped with software for personal computers (PCs), which allows the acquisition of real-time data curves through a Bluetooth connection with the detectors. Two out of the three devices were placed on the patient’s arms and connected to a PC through the software RadSight v2.23.5.7785, which operates with a Bluetooth connection to visualize real-time DR curves and acquire the data.

### Radiopharmaceuticals injection procedure

PET radiopharmaceutical injections were performed by nurses with more than 10 years of experience in the nuclear medicine field. The injection procedure lasts approximately 10 minutes, firstly the bolus of radiopharmaceutical is injected in a few fractions of a second using a lead-shielded syringe, subsequently, a saline infusion is administered through an intravenous line, which may last up to 10 minutes. Generally, the area of injection is the antecubital fossa. Depending on the patient’s anatomy it can vary along all the patient’s arms, from hand to shoulder. All PET/CT studies were performed on a whole-body PET/CT scanner (Discovery MI, GE Medical System, Milwaukee, WI, USA). Each patient is injected with 2–3 MBq/kg of PET radiopharmaceutical and the examinations are performed after a time depending on the specific radiotracer.

Injections of therapeutic radiopharmaceuticals (^177^Lu- DOTATATE) were performed systematically (i.v. injection) by using a slow infusion system, as reported by Cremonesi et al. [27].

The radiopharmaceutical has a final volume of around 25 ml administered over a time of 10–30 min via an indwelling catheter, placed under ultrasound guide, to ensure safe intravenous administration and to prevent paravascular infiltration [28].

The infusion started by allowing saline solution to drip into the vial containing the radiopharmaceutical by gravity, thus forcing the radiopharmaceutical out of the vial to the patient i.v. port. This method used for Peptide Receptor Radionuclide Therapy (PRRT) therapies was originally developed to avoid the handling of syringes, and hence to reduce the radiation burden to the physician [27]. The duration of the infusion may depend on clinical requirements. The infusion termination was checked by measuring the dose rate outside the vial. Medical staff are responsible for the slow IV administration.

The specific procedure and radiation protection devices used during administrations were previously described by Grassi E et al. [4].

**Fig 1:**
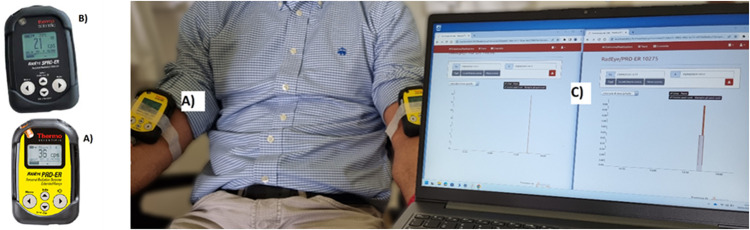
Experimental set-up. The image shows the measurement set-up used to acquire DR-time curves during diagnostic administrations. **A)** The RadEye PRD-ER device. **B)** The RadEye SPRD-ER device. **C)** RadSight software connected through bluetooth with the devices positioned on patient arms. PC screen shows real-time curves that are recorded during administration.

**Fig 2:**
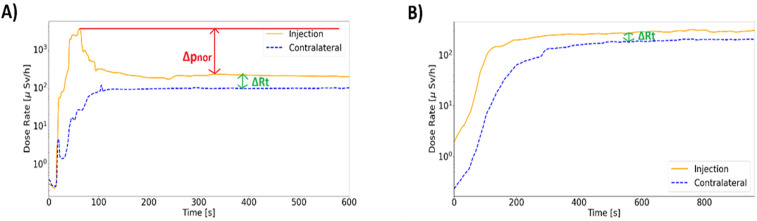
Curve acquisition. Image A) is an ^18^F-FDG ideal DR-time curve acquired during the administration; Δp_nor_ and ΔR_t_ are defined in the paragraph Measured Parameters and Data Processing. Image B) is an ideal ^177^Lu-DOTATATE DR-time curve acquired during therapeutic administration. The continuous line is the signal acquired on the injection arm; the dashed line is the contralateral signal. The calculated metric is drawn in the figure.

**Fig 3:**
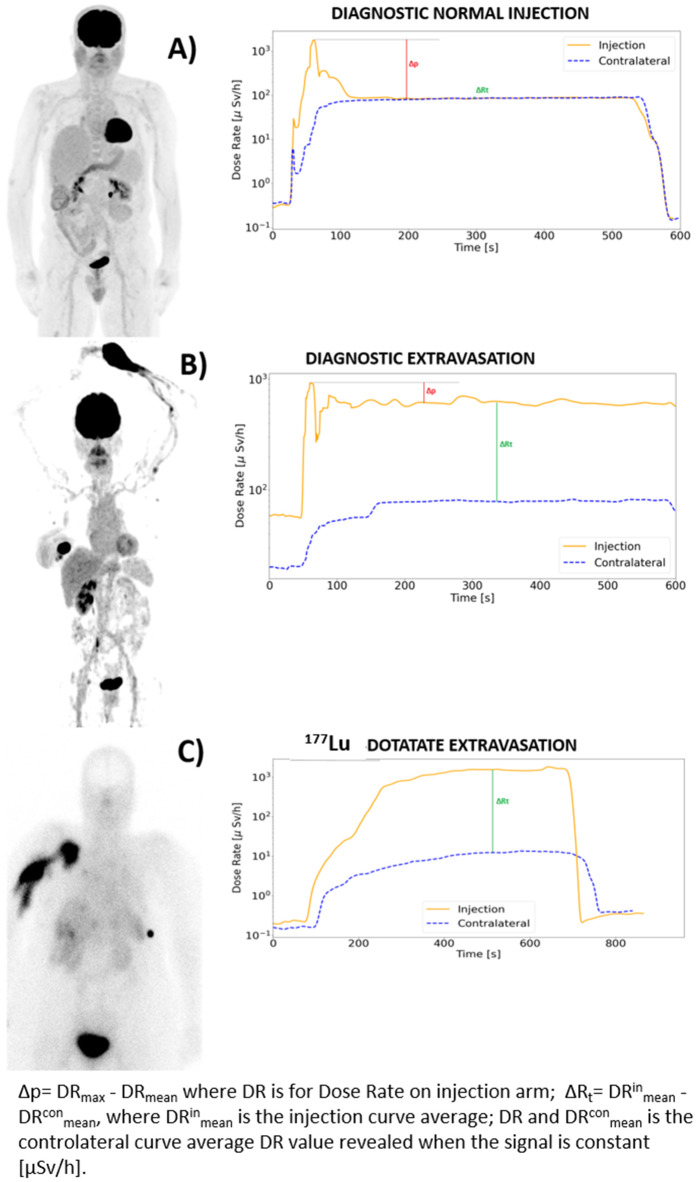
Experimental curves. A) and B) DR-time curves acquired for normal and abnormal injections for diagnostic radiopharmaceuticals with acquired PET/CT images. The metric calculated for each curve is drawn in the figure. C) DR-time curve acquired for ^177^Lu-DOTATATE extravasation event with acquired planar scintigraphy 2 hours after therapeutic administration. The metric calculated is drawn in the figure.

**Fig 4:**
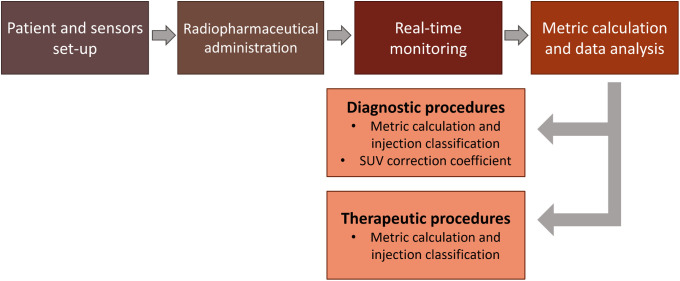
Clinical workflow. Real-time detection of extravasation events and SUV correction calculation workflow. The time required from step 1 to step 3 is 8 minutes for diagnostic injections and 15 minutes for therapeutic ones. Step 4 is different for diagnostic and therapeutic procedures. However, it is an off-line process, that requires 5 minutes with homemade software written in Python both for therapeutic and diagnostic patients.

In both diagnostic and therapeutic cases, the two detectors were secured to the patient’s arms before the start of the injection. The first device is placed approximately 5 cm proximal to the injection site, while the second is placed on the contralateral arm, making sure that their positions are almost mirrored. The sensors are connected to the PC through a Bluetooth connection using the RadSight software. The measurement setup is reported in Fig 1. Both sensors were left in place until the end of the IV administration and all DR data can be visualised in real-time on the PC monitor. The detectors were set for collecting data at 1-second intervals, and the DR time has been saved in CSV format to be analysed later. An example of a DR-time curve visualised on the PC monitor and elaborated by homemade software is shown in Fig 2 and 3. The study workflow is shown in Fig 4.

### Measured parameters and data processing

DR-time curves, acquired by monitoring the injection of diagnostic and therapeutic radiopharmaceuticals, show similar behaviours. The curve acquired on the injection arm (injection curve) reaches a maximum when the bolus goes close to the sensor. Then, the radiopharmaceutical bolus moves away from the detector, spreading throughout the body and reaching the contralateral arm. In this second phase, the curve exhibits a plateau where the signal stabilizes converging to the DR-time curve of the contralateral arm. The curve acquired on the contralateral arm (contralateral curve) rapidly increases with the spreading of the radiopharmaceutical, then reaching a plateau that converges to the injection curve signal. The main difference between diagnostic and therapeutic curves is the height of the peak; Fig 2 shows the injection curve and contralateral curve of patients injected with Lutetium concerning diagnostic curves. Based on the literature [14], the ideal trend for a correct IV administration is represented by the convergence towards the plateau of the DR-time curves of both patient arms, considering this behaviour a crucial element for establishing the presence of residual activity (A_RS_) in the injection region. When such behaviour occurs, it is possible to exclude the presence of extravasation. The same assumption was used in this study to evaluate the behaviour of our DR-time curves.

Based on our previous experience [23], we evaluated that an acquisition time of 8 minutes for PET exams is enough to reveal the presence of an extravasation event. The following metric was adopted:

- DR^in^_max_: injection curve maximum DR value that corresponds to the passage of radioactive bolus [µSv/h];
- DR^in^_mean_: injection curve average DR value after the measured signal reached a plateau [µSv/h];
- t*: time needed for the DR^in^ value to reach a plateau [sec];
- Δp^in^: it is the difference between DR^in^_max_ and DR^in^_mean_ (DR^in^_max_ - DR^in^_mean_);
- Δp^in^_nor_: it is the Δp^in^ value normalized for the injected activity (A_inj_) and DR^in^_max_ (Δp^in^/(DR^in^_max_ * A_inj_)).
- ΔR_t_: it is the difference between DR^in^_mean_ and DR^con^_mean_, where DR^con^_mean_ is the controlateral curve average DR value after the measured signal reaches a plateau [µSv/h].

For patients injected with lutetium the parameters Δp^in^ and DR^in^_max_, can not be considered because they are not representative of possible extravasation events. The main difference between normal and extravasation curves is shown by the relative difference between injection and contralateral DR-time curves, for this reason, only the ΔR_t_ parameter was calculated and evaluated. Also, the injection and contralateral curves were acquired over 15 minutes, because the therapeutic injection procedure is slower than the diagnostic one.

### PET diagnostic curves data analysis

The curves were exported as CSV files from the RadSight software and analysed by extracting the parameters described in the previous paragraph with a homemade algorithm written in Python v3.7.9. The curves were pre-processed cutting the signal one minute before and seven minutes after the injection peak (DR^in^_max_). A Gaussian filter was applied to the curves starting from 1 minute after the peak to reduce noise in the plateau region and prevent smoothing of the peak.

For each patient, the algorithm identifies values of DR^in^_max_, DR^in^_mean_, t*, and calculates ΔR_t_ and Δp^in^ and Δp^in^_nor_ parameters. The condition that must be satisfied to guarantee signal stability is that the curve value at two consecutive instants (i, i + 1) is not greater than 15 µSv/h for at least 60 consecutive seconds. The first point that satisfies this condition is considered the plateau starting point. We established the 15 µSv/h threshold in the previous work through curves statistical analysis [23].

With the help of nuclear medicine physicians, we classified the injection based on normal and extravasation cases basing the evidence on PET/CT images. The different administration cases were studied through the curves’ behaviour, correlating our metric’s parameters with injection type. We considered the statistical uncertainties of the devices corresponding to the relative error on measured DR which is approximately 10%.

Finally, we identified a statistical threshold on Δp^in^_nor_ and ΔR_t_ parameters that allows us to distinguish operationally extravasation cases from normal cases, with a focus on more severe extravasation cases that required a re-injection. The threshold was identified using a logistic regression calculated using Matlab v. R2021b.

### Radioligand therapy curves data analysis

The curves were exported as CSV files from the RadSight software and analysed by extracting the parameters described in the previous paragraph with a homemade algorithm written in Python v3.7.9, as for diagnostic curves. The curves were pre-processed by cutting the signal two minutes before and 10 minutes after the injection peak (DRinmax). A Gaussian filter was applied to the curves starting one minute after the peak to reduce noise in the plateau region and prevent smoothing of the peak.

For each patient, the algorithm identifies values of DR^in^_mean_, t*, and calculates ΔR_t_ parameters. We calculated and evaluated only one parameter because the injection curve wasn’t characterized by a peak. As stated before, the condition that must be satisfied to guarantee signal stability is that the curve value at two consecutive instants (i, i + 1) is not greater than 15 µSv/h for at least 60 consecutive seconds. The first point that satisfies this condition is considered the plateau starting point. The method used to establish the 15 µSv/h threshold is described in the previous work [23].

With the help of nuclear medicine physicians, we classified the injection into normal and extravasation cases. We confirmed the occurrence of one extravasation event thanks to single photon emission tomography/computed tomography (SPECT/CT) imaging acquired specifically to evaluate the entity of the extravasation. Since we have few acquired cases in the therapeutic field, we have conducted qualitative evaluations rather than quantitative ones, to demonstrate that our validated operational method in diagnostics can also be used in the therapeutic field with beta emitter radiopharmaceuticals.

### Extravasation regions and dose estimations for diagnostics and radioligand therapy

We estimated the residual activity (A_rs_) in the extravasation region from the images to evaluate its impact on local tissue in terms of absorbed dose and to estimate the SUV correction for PET scans. All the identified extravasation areas in diagnostics were segmented on PET images choosing a 3-D threshold technique using Velocity 3.2 (Varian Medical Systems, Palo Alto, USA), extrapolating the mean activity concentration values [MBq/ml] from the identified VOI.

Different approaches were proposed to segment the volumes most suitable in case of extravasation [9,20,21,29]. Following the strategy proposed by Tylski [20], and on the results obtained from our previous work [23], we segmented a volume with a threshold corresponding to 10% (Th10) of the maximum intensity uptake voxel values in the extravasated areas.

The volume as defined above is representative of the uptake volume identified by the physician in the clinical imaging.

Regarding the quantification of activity in SPECT images, the previously calculated calibration factor of the scanner for ^177^Lu was 11.75 cps/MBq. It enables the conversion of the counts provided by SPECT imaging into activity within the volume. Since the identified activity in the SPECT volume corresponds to the activity in the extravasation area at the time of acquisition of the scan, this activity value was decay corrected considering the physical half-life of ^177^Lu to obtain the activity present at the time of infusion, A_rs_.

In both cases, the absorbed doses were calculated with OLINDA version 2.3.3 (Hermes Medical Solutions AB, Sweden) using the sphere model [22], taking into account the mean activity in the extravasation and considering masses as closer as possible to the volumes of extravasations [23].

### SUV correction method for PET diagnostic procedures

SUV has the role to differentiate between normal and abnormal uptake and it is influenced by various patient-related factors, including for example VOI definition, A_in_, body size, and the time lapse between injection and image acquisition.

PET images from all participants in the study underwent analysis, with particular attention given to patients exhibiting abnormal trends in DR-time curves during administration. A senior nuclear medicine physician meticulously reviewed the PET images to identify potential extravasation cases.

In extravasation cases, SUV values were adjusted by subtracting the estimated activity at the injection site from the total A_in_ value [8,9]. This adjustment specifically quantified the impact of extravasation events. Then, we calculated the SUV_corr,coeff_ which allows estimating the correct SUV simply by multiplying the value calculated by the physician on the image by the estimated coefficient itself [23]. The coefficient is calculated from the percentage variation of the corrected SUV (SUV_%CR_), compared to the values initially estimated without considering the extravasation events (SUV_in_). Then, we studied the relation between our metric and SUV_corr,coeff._

## Results

### Preliminary data analysis

The data collected from patients who underwent PET examination and radioligand therapy were analysed separately because the number of cases and the injection procedure were very different.

Starting from PET patients, 825 out of 885 administrations were considered due to inadequate acquisition conditions. Firstly, we studied the correlation within our parameters Δp^in^_nor_ and ΔR_t_ and the following clinical parameters: age, weight, height, glucose level, and undergoing therapy with corticosteroid or chemotherapy drugs. In none of these cases, a statistically significant correlation was found (all p values were > 0.05) applying the Bonferroni-Holm method through RStudio 2022.07.2, confirming what we found in our previous study [23].

According to our prior investigation [23], we observed different injection and contralateral curves’ trends in extravasation and normal cases. Compared to the entire sample subject of the study, 772 patients reported a normal trend of the DR-time curves, confirmed by PET imaging. Instead, 53 patients showed abnormal trends considering both DR-time curves and their related metrics analysis; PET imaging confirmed the presence of an extravasation event.

Regarding therapies with ^177^Lu-labelled compound, DR-time curves of patients who underwent therapy with ^177^Lu show similar behaviour to curves acquired using diagnostic radiopharmaceuticals when they reach a stable value. For these patients, the acquisition lasts 15 minutes, because of the different injection modalities. For these curves, only the ΔR_t_ parameter was calculated, because the injection curve doesn’t show the typical peak revealed in diagnostic administration.

Of the 15 patients, who underwent therapy with ^177^Lu-labelled compound, only one patient had an extravasation event, so it wasn’t possible to establish a correlation with clinical parameters.

### Characterization metrics for PET diagnostic procedures

Considering our previous experience [23], no correction factors were used to normalise the readings of the instruments.

Based on the results found in the previous study, we decided to classify all events previously defined as lymphatic retention as normal events, as they had no clinical impact on image interpretation and diagnosis. In addition, we normalised Δp^in^ to activity as well as to the peak. We made this decision considering the strong dependence of peak height on administered activity, which could lead to a misinterpretation of results. In the previous work, due to a limited number of patients, this dependence had not been observed.

Starting from ΔR_t_ analysis, we calculated this parameter using a mean value over the DR curve plateau, instead of calculating it at specified time intervals. This decision was made to create an operational quantitative parameter that accounts for the overall trend of the contralateral curve compared to the injection curve, not just specific time points. The median ΔR_t_ value with its 95% range for normal and extravasation cases is 46 [13–103] µSv/h and 279 [23–13473] µSv/h, respectively. Unpaired Two-Samples Wilcoxon rank sum test was performed using Rstudio 2022.07.2 to confirm the significant statistical difference between ΔR_t_ of the two classes, resulting in a p-value less than 0.05. The obtained values confirm the results achieved in the previous study, validating a completely different trend in normal cases compared to extravasation cases in the plateau region of the injection and contralateral curves.

**Fig 5:**
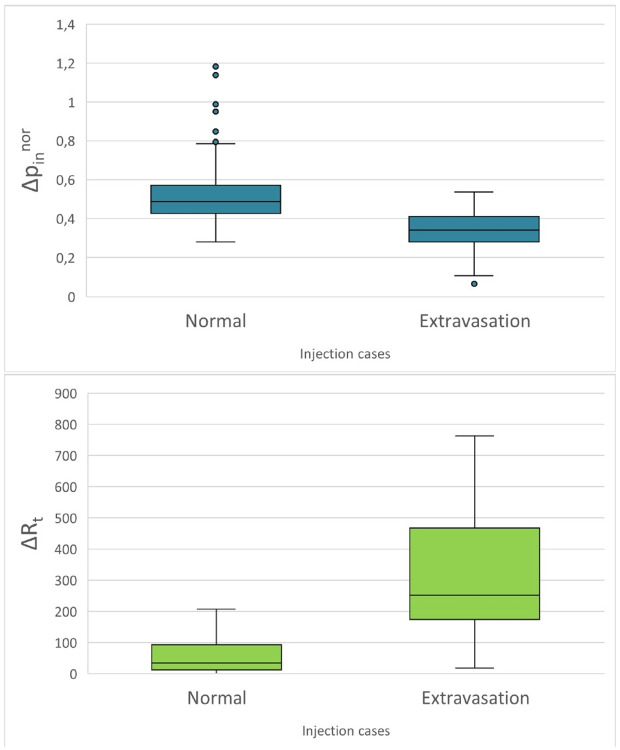
Statistical results for diagnostics. Boxplot of ΔpinNOR and ΔRt for the two different classes of injection. The upper figure shows a box plot with the median value and interquartile range of ΔpinNOR for the two injection classes, and the lower figure shows a box plot with the median value and interquartile range of ΔRt for the two injection classes.

Proceeding with Δpinnor analysis, Δpin value was normalized for peak and activity value to reduce the dependence on peak height. The median Δpinnor values with the 95% normal and extravasation administration range are 0.5 [0.42–1.18] and 0.34 [0.29–0.53], respectively. The Δpinnor values belong to statistically different populations according to the unpaired Two-Samples Wilcoxon rank sum test performed using Rstudio 2022.07.2 (a p-value less than 0.05). Also, for this parameter, we confirmed different trends for normal and extravasation cases within the peak and plateau regions of the injection curve. The obtained statistical results are shown in Fig 5.

**Fig 6:**
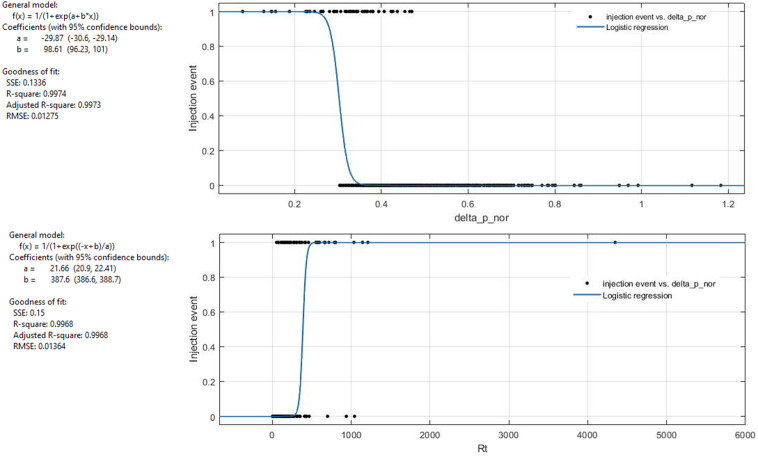
Logistic regression for diagnostics. An operational threshold (0.5 of the logistic curve) was identified to distinguish between normal injections and extravasation events. The upper curve refers to ΔpinNOR, the lowest to ΔRt.

Finally, we used logistic regression to set a threshold on Δpinnor and ΔRt parameters to identify extravasation events using Matlab v. R2021b. The threshold values at 0.5 of the logistic curves are 0.3 and 388 µSv/h for Δpinnor and ΔRt respectively with R2 of 0.99 (Fig 6). The results found on the thresholds applied to the metrics for identifying cases of extravasation confirm the hypothesis made in our previous work. It is possible to identify cases of extravasation using a single detector placed on the injection arm, by calculating the Δpinnor and applying the found threshold.

### Characterization metrics for radioligand therapy

The number of acquired patients administered with therapeutic radiopharmaceuticals is limited compared to patients who underwent PET/CT diagnostic examination. It may be expected since the number of PET studies acquired per year is much higher than radioligand treatments with ^177^Lu-labelled compound. Therefore, the calculated metric parameter has been analyzed to demonstrate that the monitoring method described for patients undergoing diagnostic acquisition is also applicable to patients administered with commonly used therapeutic radiopharmaceuticals.

**Fig 7:**
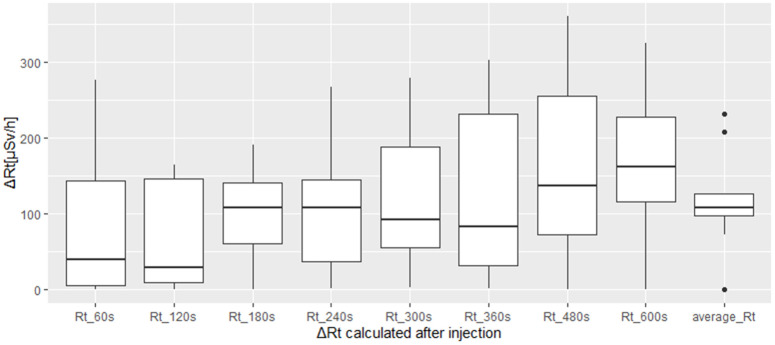
Statistical results for therapy. The figure shows the trend of ΔRt with time for normal injection cases in therapy.

For ΔRt analysis, we decided to calculate this parameter both using a mean value over 10 minutes of acquisition of the DR curve plateau and calculating it at specified time intervals: 60s, 120s, 180s, 240s, 300s, 360s, 480s, 600s after the beginning of the injection, similarly to [23]. A Kolmogorov-Smirnov test was used to statistically study the difference between ΔRt calculated at each time interval and the ΔRt value calculated over the mean plateau value over 10 minutes acquisition (ΔRtmean). The resulting p-value for each test is higher than 0.05, so it’s possible to evaluate the curve behaviour using only the ΔRtmean without evaluating the curve at each time interval, the results are graphically shown through boxplots in Fig 7. The median ΔRtmean value for normal cases, along with its interquartile range, is 108 [80–200] µSv/h. In the case of extravasation, we only acquired a single curve, so we report a single ΔRtmean of 828 µSv/h, without an interquartile range.

Performing a statistical test to evaluate the difference between the two classes of injection isn’t possible because we don’t have a distribution for extravasation cases. However, observing curves in Fig 7 it is possible to assume that the trend is like diagnostic curves for ΔR_t_^mean^. Also, ΔR_t_^mean^ median values percentage difference between the two classes is 87%.

Finally, ΔR_t_^mean^ value calculated over the first 10 minutes acquisition isn’t statistically different (Kolmogorov-Smirnov test’s p-value> 0.005) from ΔR_t_^mean^ value calculated over the first 5 minutes after injection (ΔR_t_^mean^_5_) with median value and interquartile range equal to (99 ± 54) µSv/h for normal injections and (759 ± 0) µSv/h for extravasation.

### Dose estimation and extravasation impact on diagnostic and radioligand therapy procedures

In case of extravasation, we estimated the self-dose in the injection tissue area. Table 1 reports the data for patients who had severe extravasation events during diagnostic procedures according to the evaluation of nuclear medicine physicians.

**Table 1:** Dosimetry results for diagnostics.

Patient ID	Volume (cm^3^)	Mean activity concentration (Bq/ml)	A_RS_(MBq)	Dose factor (mGy/MBq)	Self-dose (mGy)	Δp^in^_nor_	ΔR_t_^mean^ (µSv/h)
04_05_18F_07 *	25.7	88286	3.3	16.5	54.8	0.15	803
17_07_23_18F_05	10.1	111222	1.6	40.1	65.9	0.26	276
05_09_23_18F_02	52.9	56897	4.4	8.4	37	0.47	70
06_09_23_18F_05 *	36.2	941451	49.9	6.84	341.4	0.12	2432
25_10_23_18F_10	4.3	387552	2.4	90.8	221.6	0.45	229
26_10_23_18F_10	13.3	623173	12.1	30.9	375	0.22	1571
15_11_23_18F_04 *	24.8	616698	22.4	17	381	0.08	13473
22_09_18F_04 *	14.9	856960	18.7	27.6	517	0.1	667
03_10_18F_06	15.4	430253	9.7	26.8	261	0.32	578
07_10_18F_04	24.9	249525	9.1	17	155	0.28	272
15_03_18F_10 *	14.9	450383	9.8	27.7	272	0.08	4346
17_01_68Ga_02	16.3	296721	7.1	41.1	291	0.32	485

Detailed dosimetry results for more severe extravasation cases, including the segmentation volume, the average activity concentration (Bq/ml), the A_RS_ (Residual activity in the extravasation region)

in the injection site (MBq) decay corrected, the dose factor (mGy/MBq), the calculated patients’ self-dose (mGy) and the metric parameters calculated for the patients (Δp^in^_nor_is the Δp^in^ value normalized for the injected activity, ΔR_t_ is the difference between DR^in^_mean_ and DR^con^_mean_). The patients with * symbol were re-injected.

The effect of extravasation is evaluated through the calculation of A_rs_ in the extravasation region using the 10% threshold method [20,23]. Table 1 shows self-absorbed doses in the extravasation area, with a maximum value of 517 mGy (0.517 Gy). Using the same threshold method, we evaluated the self-absorbed dose in the extravasation area of a patient injected with therapeutic radiopharmaceutical, results are shown in Table 2 with a self-absorbed dose of 11.6 Gy.

**Table 2:** Dosimetry results for therapy.

Patient ID	Volume	Counts	A_RS_(MBq)	Dose factor (mGy/MBq)	Self-dose (Gy)
13_11_23_177Lu	342,5	818	286	40.7	11.6

Detailed dosimetry results for therapeutic extravasation case, including the segmentation volume, counts in the segmented volume, the A_RS_ (Residual activity in the extravasation region) in the injection site (MBq), the dose factor (mGy/MBq), the calculated patients’ self-dose as product between A_RS_ and dose factor (Gy).

**Fig 8:**
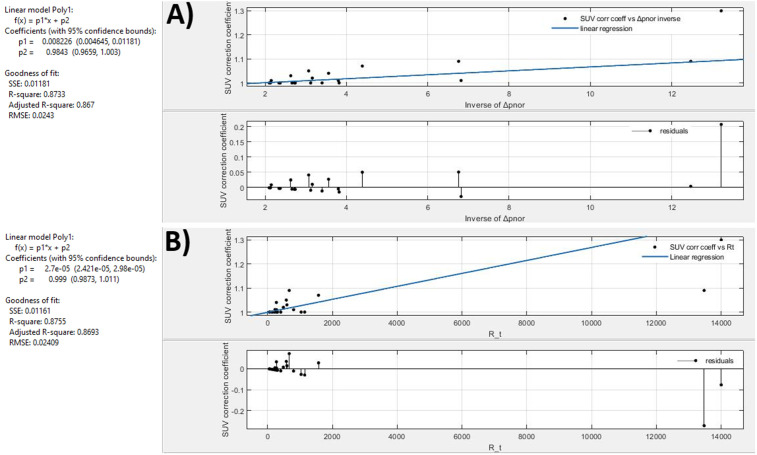
SUV correction factors. Fitted curves of the inverse of Δp^in^_nor_ (figure A) and ΔR_t_ normalized (figure B) versus SUV correction coefficient. Ideal cases were associated with a cumulative point with an SUV correction coefficient equal to 1.

We evaluated the impact of extravasation events on SUV calculation through A_rs_, correlating the SUV_corr,coeff_ with our calculated metric. SUV_corr,coeff_ is a multiplicative coefficient, which allows estimating the correct SUV simply by multiplying the value calculated by the physician on the image by the estimated coefficient itself. The SUV_%CR_ ranges between 0.004% − 30.1%. The SUV_corr,coeff_ was calculated putting it equal to 1 for normal or non-clinically relevant abnormal injections, while we calculated it as 1 + SUV_%CR_ for extravasation cases. In our previous work, presented as a feasibility study, we found an exponential correlation between SUV_corr,coeff,_ and ΔR_t_ and the inverse of Δp^in^_NOR_. In this work, we added new extravasation cases to the curve, excluding patients with extravasation without adequate imaging (the injection site was not fully within the PET FOV). Fig 8 shows the curves created by placing on the x-axis the value of the metrics, respectively the ΔR_t_ and the inverse of Δp^in^_NOR_, and on the ordinates SUV correction factors and residuals analysis. The two graphs and their fittings were created using the Matlab v. R2021b application ‘curve fitting’. They differ from the exponential behavior found before (with a limited cohort of patients), showing a linear trend. The fitting function is the same for both metrics: SUV correction coefficient = p_1_*metric + p_2_.

The metric can be ΔR_t_ and the inverse of Δp^in^_NOR_, *p*_*1*_ and *p*_*2*_ are the coefficients of the fitting. Then, the R2 factor and the root mean square error (RMSE) were compared, to assess the goodness of fit. Both fitting curves return an R^2^ equal to 0.87 and an RMSE equal to 0.02.

## Discussion

The study aimed to confirm the feasibility and applicability of our method of monitoring injections in nuclear medicine, which has already been studied and presented in a previous work [23]. Portable dosimeters were applied to the patient’s arms to detect and characterise the presence of anomalies or extravasation events. This method was also adopted in radioligand therapies. The monitoring time is lower than 10 minutes for diagnostic injections and 15 minutes for therapeutic administrations. The use of a large cohort of patients in diagnostics allowed us to improve the metric investigated in the previous paper [23]. Furthermore, the metric was adapted to treatments, allowing extravasation events to be identified promptly and allowing the user to stop the administration.

The obtained results confirmed the possibility of estimating the presence of extravasation in diagnostic administrations and evaluating SUV correction coefficient using a single detector placed on the injection arm.

Our method allows real-time monitoring during administration, together with extravasation identification. In our patient cohort, 53 out of 885 abnormal diagnostic administrations, and 1 out of 15 abnormal therapeutic administrations were identified. All of these abnormal administrations were confirmed by CT/PET and SPECT/CT acquisition.

This work aimed to demonstrate the feasibility of the method presented in our previous paper, adding statistical significance to the former analysis. In our first study, we analysed the feasibility of this monitoring method using two portable spectrometers that allowed only wired data export and we observed only 9 abnormal administrations out of 69 patients injected with ^18^F-FDG. Also, the statistical analyses were conducted only on 4 extravasation events. In this study, we focused on acquiring better statistical significance, through the analysis of 1770 diagnostic DR-time curves (885 DR curve for the injection arm and 885 for the contralateral arm) of patients injected with ^18^F-FDG, ^68^Ga-PSMA-11, and ^68^Ga-DOTATOC. The trend of the curves is shown in Fig 2 and Fig 3. This monocentric study allowed to identify the trend of the two different classes of administration (i.e., normal and extravasation) similar to those already mentioned in the literature [11,13,14,16]. We have decided to exclude the class of lymphatic retentions mentioned in the previous article [23] from the study. This decision was made in agreement with the nuclear physicians at our institution, who consider these events insignificant from both clinical and diagnostic perspectives.

Although DR-time curves of IV administrations showed similar behaviours in terms of DRinmax, DRinmean, t*, in general, two different mean trends were noted (Fig 3), characterised by ΔR_t_ and Δp^in^_NOR_ analysis.

According to the obtained results, ΔR_t_ values are significantly different (p < 0.05) for normal and extravasation classes of injection, with median value and 95% range of 46 [13−103] µSv/h and 279 [23−13473] µSv/h respectively. Also, Δp^in^_NOR_ values are significantly different (p < 0.05) for the two injection classes, with median value and 95% range of 0.5 [0.42–1.18] for normal cases and 0.34 [0.29–0.53] for extravasation cases. Operationally, it is helpful to define a threshold value for the two calculated parameters. The threshold values at 0.5 of the logistic curves are 0.3 and 388 µSv/h for Δp^in^_NOR_ and ΔRt, respectively, with R2 of 0.99. In the previous study, it was impossible to define the threshold values identified in this work due to insufficient data. However, in the present study, we collected data from a total of 885 patients, allowing for a more robust analysis and a more precise determination of these thresholds. It is worth highlighting that the threshold identified for parameter Δp^in^_NOR_ is sufficiently robust to allow the use of a single detector instead of two, relying solely on this parameter for the identification of extravasation cases. Notably, all Δp^in^_NOR_ values below the threshold were reinjected, as can be seen from Table 1. Regarding therapeutic administrations, it wasn’t possible to perform quantitative analyses reaching adequate statistical significance due to the limited available patient sample (15 patients, 1 extravasation). From the obtained results, we can affirm that this method can also be applied to therapy cases, using ΔR_t_ as a metric. Furthermore, ΔR_t_mean calculated over the first 10 minutes of acquisition isn’t statistically different (Kolmogorov-Smirnov test’s p-value> 0.005) from ΔR_t_mean value calculated over the first 5 minutes after injection (ΔR_t_mean5) with median value and interquartile range equal to (99 ± 54) µSv/h for normal injections and (759 ± 0) µSv/h for extravasation (Fig 7). It could be concluded that 5 minutes of post-injection monitoring may be sufficient to identify an extravasation event and promptly stop the injection.

All the diagnostic radiopharmaceuticals’ extravasation cases show a percentage variation on SUV that agrees with data in the literature, with a maximum percentage variation of 30% [9].

In our previous work [23], we observed an inverse proportionality between Δp^in^_NOR_ and SUV_%CR_, and a direct proportionality between ΔR_t_ and SUV_%CR_. From our previous results, we assumed that the Δp^in^_NOR_ parameter could intervene in the evaluation of the entity of the extravasation phenomenon, returning a priori a percentage directly linked to the A_RS_.

Fig 8 shows a linear trend of the SUV_corr,coeff_ in function of ΔR_t_^NOR,^ and the inverse of Δp^in^_NOR_. The fitting function found in this study is different from that found in the previous work [23]. The reason relies on a bigger number of patients acquired and analyzed in our previous research (from 4 patients to 22). Recalling that Δp^in^_NOR_ is a parameter that is calculated on the curve of the injection arm, the fact that the SUV correction factor has the same trend as a function of the inverse of Δp^in^_NOR_ and ΔR_t_^NOR^, permits to the observation that it is sufficient to acquire only the time-dependent DR curve of the injection arm to characterise the presence of anomalies or extravasation events in real-time. This finding is crucial, considering the possibility of recognizing and monitoring extravasation events using a single sensor placed in areas different from the antecubital fossa (e.g., in the foot) due to difficulties in accessing the blood vessel. In addition, the use of a single detector is more comfortable for patients and requires less data processing time.

The linear correlation identified between the SUV correction factor and the inverse of Δp^in^_NOR_ allows to calculate the SUV correction before image acquisition. In this way, clinicians can evaluate the need to integrate the injected activity to acquire adequate PET/CT imaging.

Alongside analysing the trends in DR-time curves, we explored the tissue self-dose in the injection site resulting from extravasation. We have identified 3 main works in the literature that were dedicated to dose estimation of extravasations of radiopharmaceuticals [19–21]. We believe that assessing the dosimetry of extravasation events can help clinicians identify patients who might experience adverse tissue reactions. The dose calculation is affected by some limitations among which is the use of the physical half-life of the radiopharmaceutical in the extravasation area instead of its effective clearance half-life. We chose this approach cautiously, given the absence of a patient-specific curve detailing the radiopharmaceutical’s kinetics. Moreover, the dose factors we used are based on a spherical volume and provide only an approximation of the anatomical area segmented on patients’ images. Employing a voxel-based method with a more sophisticated dose calculation model could help clarify the limitations of our current approach. Nevertheless, despite these constraints, our dose estimates align with the lack of observable effects or mild adverse effects, such as redness and swelling at the injection site. In the case of lutetium extravasation, thanks to our monitoring, we were able to stop the injection early, by minimising the out-of-vessel dose. The self-dose in the segmented volume of 11.6Gy is in agreement with the mild side effects observed such as swelling and redness, as observed also by Tylski P et al. [20]. In addition, SPECT/CT acquired one day after the injection showed no persistence of the radiopharmaceutical at the injection site.

The described sensors would enable healthcare professionals administering injections to detect extravasations within the first few minutes post-injection. Their lightweight design and user-friendly interface support further development of this system, promising a straightforward method for screening and monitoring IV administrations. Real-time DR detection on the patient’s arms, combined with device reading monitors and wireless PC connectivity, facilitates quick identification of extravasation events, allowing the identification of patients that need a re-injection through our calculated metric. Moreover, this monitoring method can be adapted for radiopharmaceutical therapy, allowing for the immediate interruption of the injection to minimise intracutaneous self-dose at the extravasation site and to reduce patient risk.

Additionally, characterising the Δp^in^_NOR_ parameter in diagnostic exams provides the opportunity to use a single sensor in the injection area for identifying extravasation events. This also enables the estimation of the SUV_corr,coeff_ in PET diagnostic exams.

## Conclusions

Optimising the process of radiopharmaceutical administration has become increasingly important, with a focus on promptly detecting extravasation events and other anomalies to minimise their impact. This study, following a previous feasibility study, allowed the development of a statistically robust method for identifying cases of extravasation in diagnostics and correcting the quantitative SUV value. The method can be easily applied by healthcare personnel in real-time during administration, enabling the identification of more severe cases of extravasation and promptly proceeding with a second injection. The identified method for applying a correction factor to the SUV is useful for the accurate interpretation of PET/CT images and diagnosis by nuclear medicine physicians. Regarding radioligand therapy, this initial study allowed us to characterize the injection curves of ^177^Lu-labelled compound. We were able to verify a difference between the curve of a normal case and an extravasation case; however, due to the lack of a large statistical sample, this difference is not statistically significant.

The absorbed doses in extravasation regions are consistent with those found in the literature and highlight the importance of prompt intervention in cases of extravasation.

## Abbreviations

A_in_: Injected activity
A_RS_: Residual activity in the extravasation region
CT: Computed Tomography
DR: Dose Rate
DR^in^: Dose Rate time curve acquired on the injection arm
DR^con^: Dose Rate time curve acquired on the contralateral arm
DR^in^_max_: Maximum Dose Rate value acquired on the injection arm
DR^in^_mean_: Average Dose Rate value acquired on the injection arm after the measured signal reached a plateau
IV: Intravenous
PET: Positron Emission Tomography
SD: Standard Deviation
SUV: Standardized Uptake Value
SUV_%CR_: Percentage variation of the correct SUV value
SUV_in_: Initially estimated SUV’s value without considering the extravasation event
t*: Time needed for the DR^in^ value to reach a plateau
VOI: Volume Of Interest
